# The Costs and Benefits of Choice: Family Managers in Directly Funded Home Care

**DOI:** 10.1093/geroni/igab046.1398

**Published:** 2021-12-17

**Authors:** Lisette Dansereau, Christine Kelly, Katie Aubrecht, Amanda Grenier, Allison Williams

**Affiliations:** 1 University of Manitoba, Winnipeg, Manitoba, Canada; 2 St. Francis Xavier University, St. Francis Xavier University, Nova Scotia, Canada; 3 University of Toronto and Baycrest Hospital, Toronto, Ontario, Canada; 4 McMaster University, Hamilton, Ontario, Canada

## Abstract

Directly funded (DF) home care, or consumer directed home care, gives program users a budget to choose their own services. Set in the Canadian province of Manitoba, our study examines the local DF program “Self and Family Managed Care”, which does not allow program users to hire and pay a family member. Incorporating a disability lens into care and aging studies, we share findings from a qualitative study based on 24 semi-structured interviews with DF users. We focus on the experiences of family managers, that is, representatives acting as a decision maker for an older adult. About half of the family managers in this study care for people living with dementia or cognitive decline. We identify two main themes: 1) service flexibility in DF reduces caregiver strain, 2) family managers tend to hire agencies rather than individuals to avoid administrative burden. Our discussion highlights the costs of DF from the perspective of caregivers as administrative burden (financial paperwork, finding workers, choosing a ‘good’ agency), and the benefits as flexibility (choosing workers, trusting workers, setting schedules, assigning work). We also consider the goals of family managers to enhance quality of life and avoid long-term residential care, in contrast to younger self-managers who desire control and autonomy. We recommend that DF programs need to reduce administrative work for users, support users in making informed choices, and find better ways to support, acknowledge and value the work of family managers and substitute decision makers.

